# The mediating role of the boredom and loneliness dimensions in the development of Problematic Internet Use

**DOI:** 10.1192/j.eurpsy.2023.1297

**Published:** 2023-07-19

**Authors:** G. Longo, L. Orsolini, U. Volpe

**Affiliations:** University Psychiatric Clinic, Department of Clinical Neuroscience/DIMSC, Università Politecnica delle Marche, Ancona, Italy

## Abstract

**Introduction:**

During the last decade, a growing digitalization allowed to implement technologies in daily life activities. Conversely, the increased use of technologies in general population, particularly in youths, facilitated the emergence of new web-based psychopathologies, including Pathological Internet use (PIU).

**Objectives:**

Our study aims at investigating the relationship between PIU and boredom as well as loneliness dimensions in youths, by also focusing on the association with the main psychopathological symptomatology (i.e., depression, anxiety and stress).

**Methods:**

A nationwide population-based cross-sectional case-control study was conducted by recruiting a sample of Italian young adults (aged 18-24), using a snowball sampling strategy. After data cleaning, only 1,643 participants were selected for analysis based on age and classified according to the presence/absence of PIU/non-PIU. Linear regression analyses as well as Pearson correlation analyses were conducted to check for possible associations and correlations between PIU and stress/anxiety/depression. Subsequently, mediation analyses regarding boredom and loneliness were conducted on these relationships.

**Results:**

Participants were predominantly females (68.7%; n = 1,129). The mean age was 21.8 years (SD = 1.7), particularly ranging 20-24 years-old (88.5%; n = 1454). Around 41.7% (n = 685) of the sample declared previous psychological issues without a history of professional support (psychological and/or psychiatric), while 32.7% (n = 538) stated that they had an overt mental disorder and were currently receiving professional support. Mediation analysis demonstrated that both boredom and loneliness act as mediators in the association between PIU and depression.

**Conclusions:**

Further studies are needed to evaluate how boredom and loneliness dimensions could be managed in order to alleviate the emergence of PIU in youths with clinically relevant depressive symptomatology.

**Disclosure of Interest:**

None Declared

